# High miR-100 expression is associated with aggressive features and modulates TORC1 complex activation in lung carcinoids

**DOI:** 10.18632/oncotarget.25541

**Published:** 2018-06-08

**Authors:** Ida Rapa, Arianna Votta, Gaia Gatti, Stefania Izzo, Nicola Lo Buono, Elisa Giorgio, Simona Vatrano, Francesca Napoli, Aldo Scarpa, Giorgio Scagliotti, Mauro Papotti, Marco Volante

**Affiliations:** ^1^ Department of Oncology at San Luigi Hospital, University of Turin, Turin, Italy; ^2^ Department of Medical Sciences, University of Turin, Turin, Italy; ^3^ ARC-NET Applied Research on Cancer Centre at Department of Diagnostics and Public Health, Section of Pathology, University and Hospital Trust of Verona, Verona, Italy

**Keywords:** miRNAs, miR-100, lung carcinoids, rapamycin sensitivity, mTOR

## Abstract

**Purpose:**

Mammalian target of rapamycin (mTOR) is a promising therapeutic target in advanced lung carcinoid patients. However, the mechanisms of mTOR modulation and of responsiveness to mTOR inhibitors are largely unclear. Our aim was to analyze the expression and functional role of specific miRNAs in lung carcinoids as an alternative mechanism targeting mTOR pathway.

**Experimental design:**

Seven miRNAs, selected by bioinformatic tools and literature search, were analyzed in 142 lung neuroendocrine neoplasms (92 carcinoids and a control group of 50 high grade neuroendocrine carcinomas), and compared with mTOR mRNA expression and clinical/pathological parameters. Tissue results were validated *in vitro* in two lung carcinoid cell lines by specific RNA interference and biological/pharmacological tests.

**Results:**

Tissutal expression of five miRNAs (miR-99b, miR-100, miR-155, miR-193a-3p, miR-193a-5p) was inversely correlated with mTOR mRNA expression, supporting their role in the negative regulation of mTOR transcription. High expression of miR-100, miR-193a-3p and miR-193a-5p was associated with aggressive features and, for the former two, with shorter time to progression. In H727 and UMC11 lung carcinoid cells, miR-100 modulated mTOR RNA and TORC1 complex protein expression, positively promoted cell migration and negatively influenced cell proliferation. Moreover, miR-100 directly influenced responsiveness of H727 and UMC11 cells to rapamycin.

**Conclusions:**

MiR-100 actively participates to the regulation of mTOR expression in lung carcinoids and represents a novel candidate prognostic biomarker for this tumor type; moreover, inhibition of its expression is associated to increased responsiveness to mTOR inhibitors and might represent a novel strategy to sensitize lung carcinoids to these target agents.

## INTRODUCTION

The mammalian target of rapamycin (mTOR) is a downstream effector of PI3K/AKT kinases and acts through two complexes, mTORC1 and mTORC2. MTORC1 promotes cell growth, cell cycle progression and anabolism, such as protein and lipid biogenesis, and at the same time inhibits catabolism by blocking authophagy; conversely, mTORC2 regulates cell survival, cell proliferation and metabolism [1].

Rapamycin and its derivatives, such as everolimus, are selective mTOR inhibitors that have been shown to block mTOR modulation of cell cycle progression, angiogenesis and apoptosis in several tumor cell models [2]. Few studies in lung carcinoid cells demonstrated a significant efficacy of mTOR inhibition strategies [3, 4], and supported the current indications for mTOR inhibitors present in the most recent clinical guidelines [5, 6]; moreover, a recent prospective clinical trial indicates a significant improvement of survival in patients with progressive lung neuroendocrine tumors treated with everolimus [7].

The clinical efficacy of mTOR inhibitors in lung carcinoids – and neuroendocrine cancer in general - might be improved by identifying i) the molecular mechanisms of mTOR pathway activation in cancer cells, and - as a consequence – ii) predictive markers of response to mTOR inhibitors. Indeed, while high levels of mTOR activation have been described in lung carcinoids in “prevalence” studies [8], no data are available about the correlation between specific activation profiles and response to therapy. Data obtained from primary tumor cell cultures, only, claim that patients with high levels of mTOR activation are associated with better responses [9].

Concerning the mechanisms leading to mTOR pathway deregulation, molecular alterations in oncogenes and tumor suppressor genes that are members of the pathway were recently found in lung neuroendocrine neoplasms, but with a relatively low prevalence in carcinoids [10, 11]. Thus, it has to be hypothesized that alternative regulators are responsible for mTOR expression. Among others, miRNAs are involved in regulating the mTOR pathway in several ways, either by targeting key genes within the pathway or interacting directly with mTOR. Several microRNAs (miRNAs) have been reported to selectively modulate mTOR pathway in other tumor models [12], but never in lung carcinoids. MiR-100, miR-199a-3p and miR-99 (a and b) directly target the 3ʹ-UTR of mTOR and suppress translation of its mRNA, and were found to be deregulated in hepatocellular, ovarian and prostate cancers, osteosarcoma, and adrenocortical tumors [13–16]. MiRNAs 193a-3p and -5p have been shown to down-regulate mTOR pathway in non-small cell lung cancer [17]. Moreover, several studies investigated the efficacy of combining mTOR inhibitors with mimic miRNAs in cancer cell models, and microRNA-driven mTOR modulation might have therapeutic benefit increasing sensitivity not only to rapamicin analogs but also to different anticancer drugs such as doxorubicin, cisplatin and taxanes [15, 18, 19].

Based on the above findings, the aim of this study was to analyze in primary lung carcinoids and cell models the expression and the functional role of selected miRNAs targeting mTOR, as an alternative mechanism of mTOR pathway regulation.

## RESULTS

### mTOR gene and mTOR-targeting miRNAs expression levels are inversely correlated in tissue samples of lung neuroendocrine neoplasm, and associated with tumor histotype

The reciprocal expression of the markers investigated was checked (Table 1) and a significant negative correlation between all but two (miR-99a and miR-199) miRNAs and mTOR gene expression was observed, indicating a potential role in controlling mTOR transcription. Moreover, the expression of all miRNAs was positively correlated each other, although with a variable degree of statistical significance and pattern of association. However, TORC1 complex-associated phosphorylated forms of mTOR and p70S6K, although strongly associated each other (Spearman's R 0.28, *p* < 0.001) were not significantly associated with any of the miRNAs (all *p* values > 0.05).

**Table 1:** Reciprocal correlation among mTOR mRNA and miRNAs expression levels

	mTOR	miR99a	mir99b	miR100	miR199	miR155	miR193a-3p
**miR99a**	*r* = 0.09 *p* = 0.2						
**mir99b**	*r* = –0.2 *p* = 0.02	*r* = –0.1 *p* = 0.3					
**miR100**	*r* = –0.4 *p* < 0.0001	*r* = 0.2 *p* = 0.03	*r* = 0.2 *p* = 0.02				
**miR199**	*r* = –0.1 *p* = 0.2	*r* = 0.2 *p* = 0.1	*r* = 0.07 *p* = 0.5	*r* = 0.08 *p* = 0.4			
**miR155**	*r* = –0.3 *p* = 0.01	*r* = –0.1 *p* = 0.2	*r* = 0.2 *p* = 0.03	*r* = 0.3 *p* = 0.003	*r* = 0.5 *p* < 0.0001		
**miR193a-3p**	*r* = –0.4 *p* = 0.0004	*r* = 0.2 *p* = 0.04	*r* = 0.2 *p* = 0.01	*r* = 0.2 *p* = 0.03	*r* = 0.3 *p* = 0.009	*r* = 0.3 *p* = 0.003	
**miR193a-5p**	*r* = –0.5 *p* < 0.0001	*r* = –0.1 *p* = 0.2	*r* = 0.1 *p* = 0.08	*r* = 0.2 *p* = 0.01	*r* = 0.4 *p* < 0.0001	*r* = 0.6 *p* < 0.0001	*r* = 0.5 *p* < 0.0001

The expression levels of mTOR gene and mTOR-targeting miRNAs were clearly different between the histologic subtype of pulmonary neuroendocrine neoplasms. In pairwise comparison, the expression levels of mTOR and miRNAs were significantly higher in carcinoid tumors than in high-grade neuroendocrine carcinoma (LCNECs and SCCs, used as control groups), except for miR-155 and miR-199 that were expressed at higher levels in high-grade neuroendocrine carcinomas (all *p* < 0.01). Moreover, miR-100, miR-193a-3p and miR-193a-5p were down regulated in TC as compared to AC (*p* < 0.0001, *p* = 0.001 and *p* = 0.001, respectively); by contrast, miR-99a and mTOR were higher in TC than AC (*p* = 0.01 and *p* < 0.0001, respectively).

### High expression of miR-100, miR193a-5p and miR193a-3p is associated with aggressive disease in lung carcinoids

The association of mTOR-targeting miRNAs expression with specific tumor characteristics was assessed in the subgroup of carcinoids, only. High-grade carcinomas, that are characterized by a completely different clinical and biological behavior, were excluded from this analysis.

Over-expression of miR-100 correlated with tumor characteristics associated to a more aggressive phenotype. Indeed, (Supplementary Figure 1) miR-100 expression level was increased in patients with lymph node metastasis (*p* = 0.04), higher tumor stage (*p* = 0.004), worse disease status (*p* = 0.04), higher tumor grade (*p* = 0.0004), and presence of vascular invasion (*p* = 0.02; not shown). Similar results were obtained for both miR-193a-3p and miR-193a-5p that were expressed at higher levels in G2 vs G1 tumors (*p* = 0.006 and *p* = 0.01, respectively) and in patients with worse disease status (*p* = 0.03 and *p* = 0.01, respectively).

### High expression of miR-100 and miR-193a-3p is associated with unfavorable prognosis in lung carcinoids

**Figure 1:**
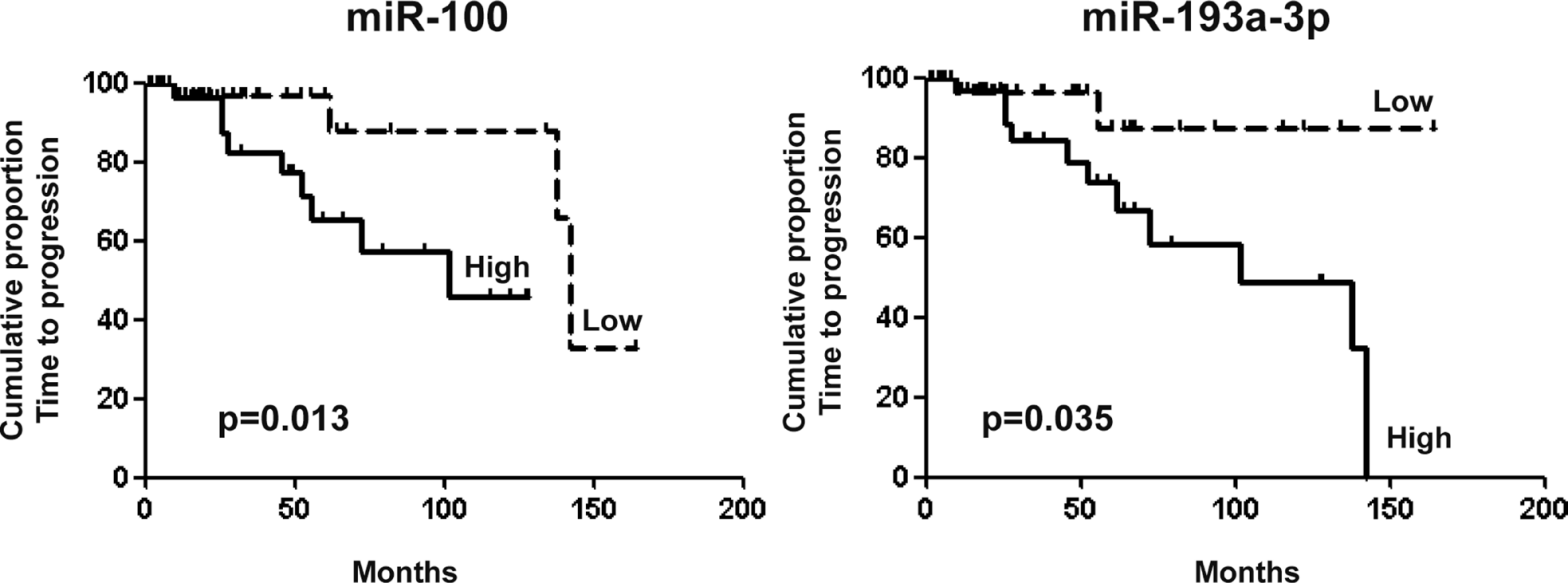
TTP analysis in lung carcinoid patients Kaplan Meier curves of survival in terms of time to progression in lung carcinoid patients segregated according to miR-100 (142 versus 101 months, log-rank 6.0, *p* = 0.01) and miR-193a-3p (undefined versus 101 months, log-rank 4.4, *p* = 0.04) expression levels.

Time to progression (TTP) in patients with low miR-100 levels was significantly longer than in patients with higher levels (142 versus 101 months, log-rank 6.0, *p* = 0.01; Figure 1). Similar results were obtained for miR-193a-3p (undefined versus 101 months, log-rank 4.4, *p* = 0.04). However, both miR-100 and miR-193a-3p failed to show any statistical significance in the specific group of atypical carcinoids, only (*p* = 0.24 and *p* = 0.48, respectively). Among clinical and pathological parameters, sex, tumor histotype, tumor grade and vascular invasion were also associated with TTP (Table 2). At multivariable Cox regression analysis, sex, histotype and grade were identified as independent poor prognostic variables.

**Table 2:** Univariate and multivariate survival analyses of time to progression in 69 lung carcinoid patients

Parameter	Univariate		Multivariate (Cox regression)	
	HR (CI)	*p*	HR (CI)	*p*
**Sex (M vs F)**	4.877 (1.6–14.6)	0.0046	0.008 (0.0–0.2)	0.003
**Age (above vs below median)**	1.537 (0. 5–4.8)	0.4633	/	/
**Histotype (AC vs TC)**	6.911 (2.2–21.9)	0.001	126.804 (5.5–2905.7)	0.002
**Rindi's Grade (2 vs 1)**	8.399 (2.3–31.0)	0.0014	0.115 (0.01–1.02)	0.048
**Size (above vs below mean)**	1.635 (0.5–5.5)	0.4263	/	/
**Clinical stage (all others vs IA/B)**	1.020 (0.3–3.4)	0.974	/	/
**Nodal status (N+ vs N0)**	1.059 (0.3–3.4)	0.932	/	/
**Vascular invasion (VI+ vs VI–)**	7.535 (1.7–32.7)	0.007	3.405 (0.9– 12.9)	0.07
**Ki-67 (≥4 vs <4)**	2.479 (0.8–8.1)	0.1314	/	/
**Ki-67 (above vs below mean)**	2.686 (0.8–9.4)	0.1207		
**miR-100 (above vs below median)**	4.507 (1.4–14.9)	0.013	0.880 (0.13– 5.7)	0.8
**miR-193a-3p (above vs below median)**	3.257 (1.07–9.85)	0.035	0.721 (0.31– 4.6)	0.7
**miR-193a-5p (above vs below median)**	2.160 (0.63–7.29)	0.2	/	/

Legend: HR: hazard ratio; CI: confidence interval; M: male; F: female; AC: atypical carcinoid; TC: typical carcinoid.

### mTOR is targeted by miR-100 in lung carcinoid cells

**Figure 2:**
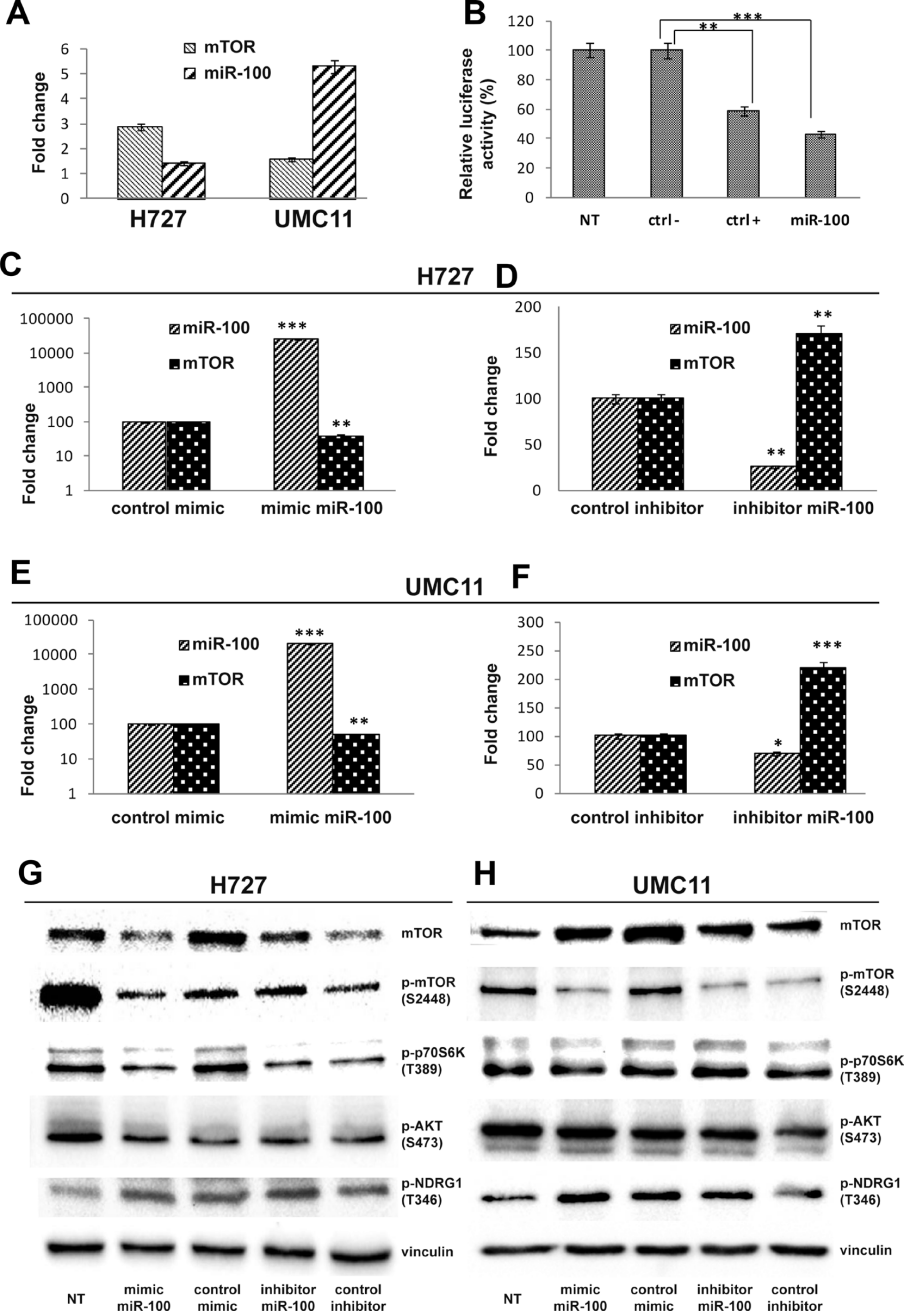
MiR-100 modulates mTOR expression levels in carcinoid cell lines (**A**) mTOR mRNA and miR-100 expression levels in H727 and UMC11 cell lines; (**B**) Luciferase assay showing reporter decreased activity after co-transfection of wild-type mTOR 3ʹUTR with miR-100 in HEK293 cells (miR-100), as in the positive control (ctrl +; see Materials and Methods for details; NT: untreated cells); (**C** and **E**) up-modulation of miR-100 by mimic miR-100 significantly reduces mTOR mRNA expression in both H727 and UMC11 cell lines; (**D** and **F**) miR-100 down-regulation by inhibitor miR-100 increases mTOR levels in both H727 and UMC11 cell lines; Western blot analysis shows that mimic and inhibitor miR-100 modulate TORC1-associated proteins but not TORC2 complex in H727 (**G**) and UMC11 (**H**) cells (see Supplementary Figure 2 for Western blot bands quantification). Each experiment was repeated in triplicate. All data in the Figure were represented as mean ± SEM. ^*^p < 0.05,^**^p < 0.01,^***^p < 0.001.

*In vitro* experiments were designed to test the specific modulation of mTOR expression by the three miRNAs showing the most significant statistical correlation in tissue samples: miR-100, miR-193a-3p and miR-193a-5p. MiR-100 was inversely associated with mTOR expression, with higher levels in low-mTOR expressing UMC-11 cells and lower levels in high-mTOR expressing H727 cells (Figure 2A). Dual-luciferase reporter assay showed that co-transfection of miR-100 significantly suppressed the activity of firefly luciferase reporter by binding the wild-type 3ʹUTR of mTOR, whereas this effect was abrogated when the HEK-293T cells were co-transfected using scrambled negative control (Figure 2B). MiR-193a-3p and -5p levels were not associated with mTOR expression neither in H727 nor in UMC-11 cells (data not shown), and luciferase activity was unaffected by miR-193a-3p or weakly suppressed by miR-193a-5p (data not shown). Therefore, miR-193a-3p and miR-193a-5p were not further considered for functional analyses. The restoration of miR-100 by means of miR-100-mimic transfection reduced the expression of mTOR at both mRNA (Figure 2C–2F) and protein (Figure 2G, 2H and Supplemental Figure 2) level in H727 and UMC11 cells, whereas the transfection of the antagonist of endogenous miR-100 (mir-100-inhibitor) increased the levels of mTOR protein and mRNA in both cells. Moreover, the same trend observed for mTOR protein in the different models in both cell lines was observed for the phosphorylated forms of mTOR and p70S6K, that are associated with TORC1 complex activation [20], whereas TORC2 was unaltered by miR100 up- or down-modulation, as demonstrated by the absence of significant changes in the phosphorylation status of AKT in S473 and of NDRG1 in T346, both known to be phosphorylated as a consequence of TORC2 activation [21].

### MiR-100 inhibition sensitizes lung carcinoid cells to rapamycin treatment

**Figure 3:**
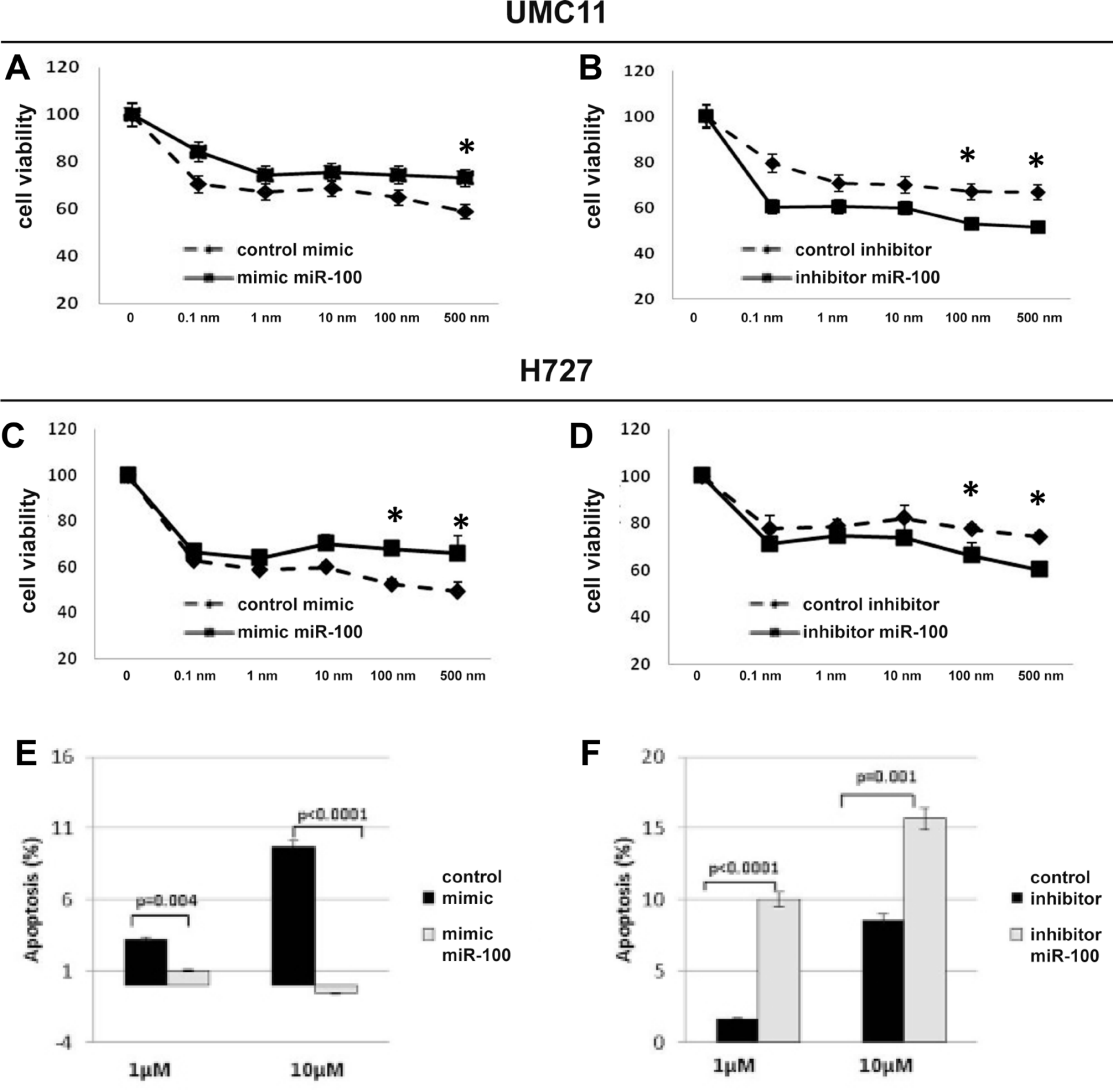
MiR-100 inhibition sensitizes lung carcinoid cells to rapamycin treatment miR-100 up-modulation reduced cytotoxic effect of rapamycin in UMC11 and H727 cells (**A** and **C**, respectively), whereas miR-100 suppression sensitized lung carcinoid cell lines (UMC11 and H727 cells, (**B** and **D**), respectively). Moreover, opposite effects on apoptosis were observed in H727 cells transfected with mimic miRNA-100 (**E**) or inhibitor miRNA-100 (**F**) under rapamycin treatment (see Results for details). Triplicate wells for each condition were examined, and each experiment was repeated in triplicate. Data was represented as mean ± SEM. ^*^p < 0.05.

To verify the hypothesis that miR-100 is functionally involved in lung carcinoid sensitivity to rapamycin, we tested H727 and UMC-11 cells in viability assays at basal conditions (transfection with mimic and inhibitor control) and after miR-100 modulation (transfection with miR-100 mimic or inhibitor). The IC_50_ value of rapamycin was higher in UMC-11 wild type (wt) cells that express miR-100 at higher levels than in H727 wt cells (1.1 μM vs 0.6 μM; *p* < 0.0001). Up-modulation of miR-100 in H727-mimic-miR-100 and UMC-11-mimic-miR-100 significantly reduced the effect of rapamycin (IC50 = 1.3 μM and IC50 = 1.5 μM, respectively) (Figure 3A and 3C) as compared with cells transfected with mimic negative control (IC50 = 0.57 μM and IC50 = 0.92 μM respectively; *p* < 0.05). In addition, down-regulation of miR-100 in H727 and UMC-11 cells transfected with inhibitor-miR-100 enhanced the effect of rapamycin at high concentrations (IC50 = 0.59 μM vs IC50 = 0.9 μM 1 and IC50 = 0.97 μM vs IC50 = 1,47 μM respectively *p* < 0.05) (Figure 3B and 3D). To further analyze the effects of miR-100 in the modulation of rapamycin-induced mTOR inhibition, we examined apoptosis in H727 cells with and without miR-100 overexpression. Apoptosis was enhanced in H727-inhibitor miR-100 cells as compared to controls (10% vs 2% at 1 μM; *p* < 0001; 15.9% vs 8.2% at 10 μM, *p* = 001) and was strongly abolished in H727-mimic miR-100 cells as compared to controls (*p* = 0004 at 1 μM and *p* < 0001 at 10 μM) (Figure 3E and 3F).

### MiR-100 modulates proliferation and migration of lung carcinoid cells

**Figure 4:**
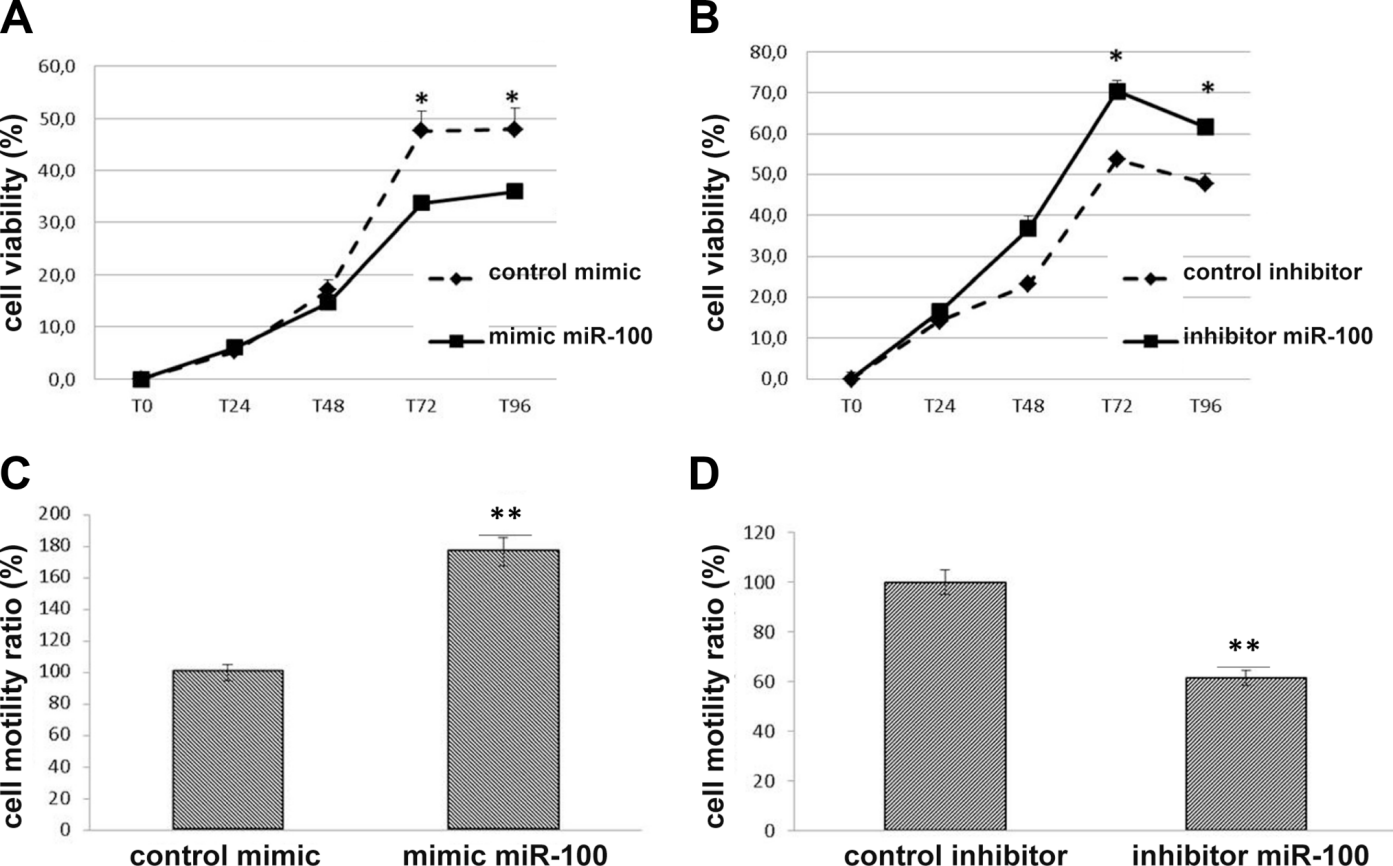
MiR-100 modulates proliferation and migration of lung carcinoid cells WST-1 assay indicated that miR-100 over-expression reduced cell proliferation (**A**) but enhanced cell motility (**C**) in H727 cells. By contrast, miR-100 inhibition increased cell proliferation (**B**) but suppressed cell motility (**D**). Duplicate wells for each condition were examined, and each experiment was repeated in triplicate. Data was represented as mean ± SEM. ^*^p < 0.05,^**^p < 0.01

To investigate additional functional mechanisms of miR-100 in lung carcinoid cells, we evaluated the role of miR-100 in the control of H727 proliferation and migration. Over-expression of miR-100 markedly decreased proliferation of H727 cells as compared to control mimic whereas the inhibition of miR-100 induced a significant increase of cell proliferation as compared to control inhibitor (Figure 4A and 4B) (*p* < 0.05). By contrast, wound healing assay showed that miR-100 over-expression led to significant enhancement of the migration of H727 cells, whereas knockdown of miR-100 significantly reduced the percentage of cellular migration as compared with inhibitor control (Figure 4C and 4D)

## DISCUSSION

**Figure 5:**
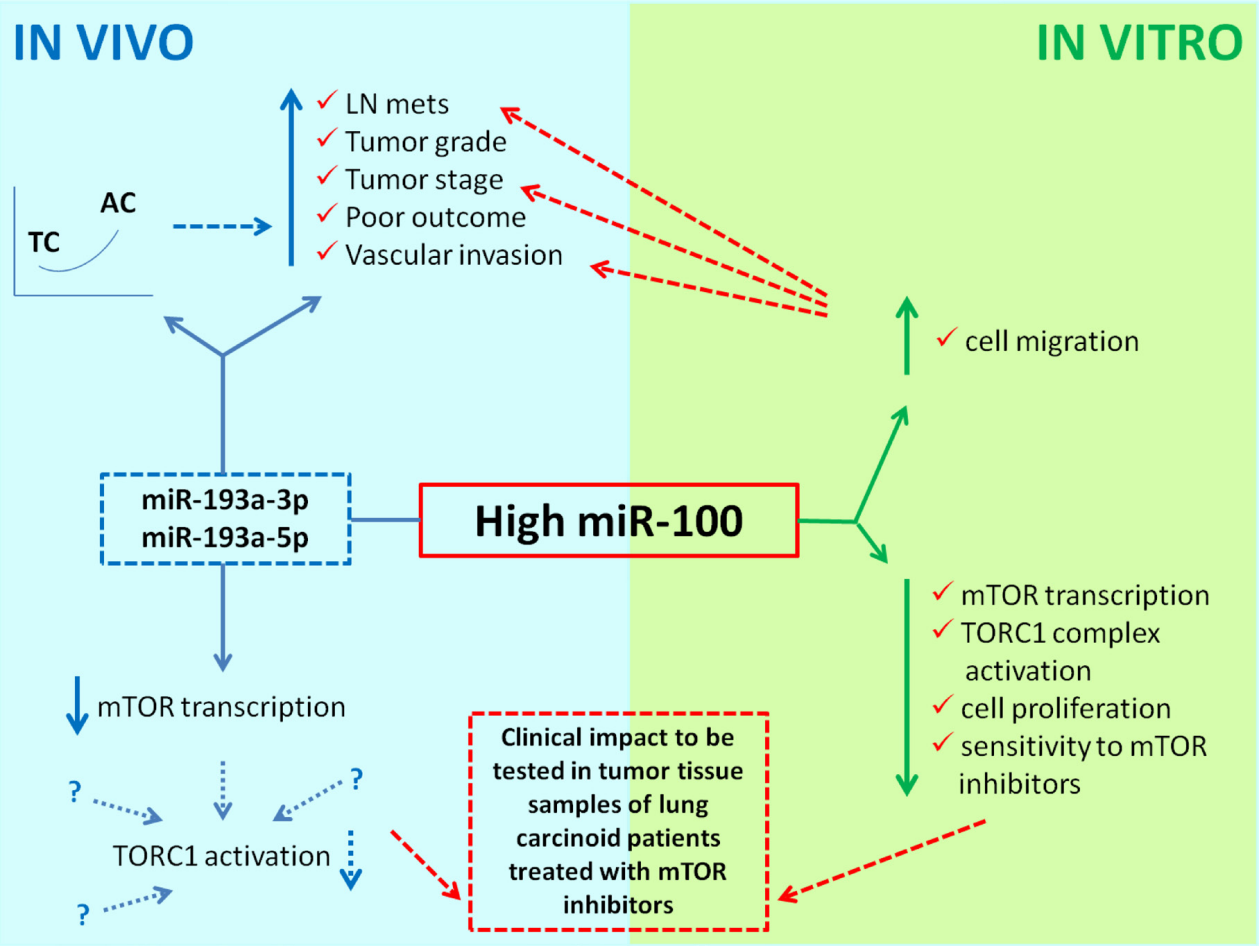
Schematic diagram illustrating the overall results of the present study Solid arrows represent conclusions based on our results. Dotted arrows illustrate possible correlations or hypotheses not directly supported but suggested by the present findings, or to be validated.

Among the strategies for targeting cellular pathways as a therapeutic tool in cancer, miRNAs have shown promising therapeutic benefit either by elevating levels of tumor suppressor or inhibiting oncogenic miRNAs. MicroRNA patterns of expression in neuroendocrine lung tumors have been already evaluated in some studies to identify novel reliable biomarkers for differential diagnosis and prognosis [22–24]. However, the role of miRNAs in targeting the mTOR pathway in neuroendocrine lung cancer is totally unexplored. The aim of this study was therefore to identify miRNAs specifically targeting the mTOR pathway in lung neuroendocrine neoplasms as well as to validate their potential functional role as modulators of mTOR pharmacological inhibition. The overall results of the present study are summarized in Figure 5.

The first goal of this study was indeed to understand whether miRNAs might be active modulators of mTOR pathway in lung carcinoids as compared to high grade neuroendocrine carcinomas. Seven miRNAs were selected and tested in tissue samples from a large series of lung neuroendocrine neoplasms in correlation with mTOR gene expression. Five miRNAS (miR-99b, miR-100, mi-R155, miR-193a-3p, miR-193a-5p) were inversely correlated with mTOR expression, thus suggesting an active interference with mTOR transcription, although they were not significantly correlated with phosphorylated forms of mTOR and p70S6K proteins, possibly as the result of the complexity of mechanisms leading to mTOR pathway activation status *in vivo*. Moreover, all five were positively correlated each other at a variable extent and were heterogeneously distributed within each tumor type category, supporting that they might interplay either as coexistent or alternative mechanisms in individual tumors in regulating mTOR gene expression. Interestingly, the high expression of miR-100, miR-193a-3p and miR-193a-5p was also associated in the group of carcinoids with specific clinical and pathological characteristics of aggressiveness, thus supporting that – either interfering with mTOR pathway or modulating tumor cells in a more complex network – they are relevant actors in controlling tumor growth and progression in these tumors.

Expression of miR-100 in human malignancies is variable and modulates tissue-specific functions being either up-regulated [25–28] or down-regulated [15, 29–33] in cancer cells. Indeed, we subsequently aimed at further supporting our findings on the biological functions of miR-100 in lung carcinoid, testing *in vitro* its role to modulate cell proliferation and invasive capacity. Our data overall support a positive control of miR-100 in invasive capacity of lung carcinoid cells which mirror the association of its over expression with higher tumor stage and positive lymph node status. By contrast, miR-100 high expression was associated with decreased cell proliferation. Even though this latter finding might be in contrast with the association with tumor grade we observed, it is worth to notice that high miR-100 expression was inversely correlated to Ki-67 proliferation index in our lung carcinoid series, even if not reaching statistical significance (data not shown). In terms of survival, high miR-100 was associated to shorter time to progression, although statistical significance was lost at multivariate survival analysis, possibly due to the limited number of events and the different distribution in tumor histotypes. Few data, mainly limited to gastrointestinal cancers [34, 35], are available about the association of miR-193a-3p and miR-193a-5p with tumor characteristics and, so far, support – as for miR-100 in the same location - an onco-suppressive role of miR-193a family members. However, as already discussed above – our data in lung carcinoids again suggest that the same miRNA might have variable effects according to the tissue where it is expressed.

The second goal of the present study was to assess the potential correlation between miRNA expression and sensitivity to mTOR inhibition. First, we validated which of the three miRNAs showing the best inverse correlation with mTOR at the tissue level (miR-100, miR-193a-3p and miR-193a-5p) directly represses mTOR expression in lung carcinoid cells, a preliminary step for further functional studies. MiR-100 was indeed the unique to be both inversely correlated at baseline with mTOR gene expression and to reduce mTOR expression at Luciferase assay. Transfection of both H727 and UMC11 cells with either mimic-miR-100 or inhibitor miR-100 confirmed the role of miR-100 in controlling mTOR gene and protein expression. A selective impairment of TORC1 complex by miR-100 was demonstrated by the specific modulation of phosphorylation of mTOR in S2448 and p70S6K in T389. More interestingly, forced up- or down-regulation of miR-100 desensitized or sensitized, respectively, both H727 and UMC11 cells to rapamycin treatment, thus showing that miR-100 modulation is able to influence response to mTOR inhibitors, and that mTOR levels are predictive of response to these agents. The major pitfall of our *in vitro* data is related to the limited availability of commercial lung carcinoid cell lines, with special reference to those growing adherent and not in suspension. However, our data support the concept that miR-100 may be responsible for inter-individual heterogeneity of mTOR expression in specific tumor types and even for the occurrence of dynamic changes of mTOR expression (and responsiveness to mTOR inhibitors) in individual patients. However, also in this context literature data are controversial, being over-expression of miR-100 either responsible of decreased or increased sensitivity to mTOR inhibitors, in lymphoblastoid [36] or ovarian cancer [15], respectively. The lack of wide correlation data between mTOR expression and mTOR inhibitor responsiveness make our results speculative, and our case series failed to be supportive of this hypothesis since no lung carcinoid patient in our cohort was treated with mTOR inhibitors at the time of this study. However, our data overall contribute to strengthen the potential promising benefit of combining miRNA-selective mTOR targeting and mTOR inhibitors as potent therapeutic tools in advanced lung carcinoids, supporting also that miR-100 expression testing might be a potential predictive biomarker of response to mTOR inhibition strategies.

In conclusion, in this study we: i) identified a set of miRNAs associated with mTOR expression and tumor characteristics in lung carcinoids; ii) demonstrated that miR-100 actively participates to the regulation of mTOR expression in lung carcinoid cells, and that its modulation influences responsiveness to mTOR inhibitors thus representing a novel target to sensitize lung carcinoid cells to this therapeutic strategy.

## MATERIALS AND METHODS

### Tumor tissue samples

A retrospective cohort of 142 formalin-fixed and paraffin embedded (FFPE) samples of surgically resected lung neuroendocrine neoplasms with available clinical and pathological characteristics, including 50 typical carcinoids (TCs), 42 atypical carcinoids (ACs), 29 large cell neuroendocrine carcinomas (LCNECs) and 21 small cell carcinomas (SCLCs), was collected from the pathology files of the University of Turin at San Luigi Hospital, Orbassano, Turin (Supplementary Table 1). All cases were anonymized by a pathology staff member not involved in the study and re-classified according to the 2015 WHO classification of lung tumors [37]. In all cases, the neuroendocrine phenotype was confirmed by positivity for at least one general neuroendocrine marker (synaptophysin, clone SP11, diluted 1:150, ThermoScientific, and/or chromogranin A, clone LK2H10, diluted 1:1500, DBS) and the proliferation index was assessed by testing Ki-67 protein expression (clone MIB-1, DakoCytomation). All diagnostic immunohistochemistry was performed using an automated immunostainer (Omnis, Dako). The proposal for lung neuroendocrine neoplasm tumor grading was also applied [38]. The Institutional Review board of the hospital approved the study (Ethics Committee Approval no. 167/2015-prot.17975, October 21, 2015).

### Bioinformatics analysis

The bioinformatics validation of selected miRNAs based on the literature and through the screening of additional mRNAs specifically targeting mTOR performed by using Web-available softwares, including miRBase and NCBI mapviewer. The predicted target genes and their conserved sites of seed region binding with each miRNAs were investigated using the TargetScan (release 5.0; http://www.targetscan.org/) and PicTAR (https://pictar.mdc-berlin.de) programs.

### RNA isolation, cDNA synthesis and microRNA expression analysis

Total RNA was isolated from tissue specimens, using miRNase miRNA isolation FFPE Kit (Qiagen), according to manufacturer's instructions. The purity and the quantity of miRNA were assessed using the BioPhotometer (Eppendorf). All samples were diluted to a final concentration of 40 ng/μl. For quantitative Real-Time PCR, cDNA was prepared using the TaqMan MicroRNA Reverse Transcription Kit (Life Technologies) as previously described [22]. Reverse transcriptase (RT) reactions containing 40 ng of total RNA were performed in a volume of 15 μl with the following conditions: 16°C for 30 min, 42°C for 30 min, 85°C for 5 min, and 4°C for 5 min. Reactions without addition of reverse transcriptase (RT− controls) were performed along with cDNA synthesis of each sample and used in subsequent procedures to control the potential genomic DNA contamination. Commercially available assays for mTOR, candidate miRNAs and housekeeping genes (Taqman, Life Technologies, Supplementary Table 2) were employed following protocols already described [22]. The amount of target miRNAs was normalized relative to the amount of RNU6B. ΔCt was calculated using the following mathematical formula: ΔCt = Ct sample- Ct control.

### Immunohistochemistry

In all samples, the expression of phosphorylated forms of mTOR (p-mTOR; clone 49F9, Ser2448, diluted 1:100; Cell Signalling) and p70S6K (p-p70S6K; clone1A5, Thr389, diluted 1:400; Cell Signalling) was assessed by means of immunohistochemistry using an automated platform (Ventana BenchMark, Roche), and scored by using the H-score (range 0–300), as previously published by our group [8].

### Cell lines

H727 and UMC-11 (lung carcinoid cell lines) and HEK-293T (human embryonic kidney cell line 293T used for luciferase assay) were from the American Type Culture Collection (ATCC). Cell lines were maintained in RPMI-1640 (H727 and UMC-11) or DMEM (HEK-293T) supplemented with 10% fetal calf serum, 2 nM glutamine, penicillin (25 U/ml), and streptomycin (25 μg/ml) (all from Sigma-Aldrich) in a humidified atmosphere containing 5% CO_2_ at 37°C.

### Cell transfections

For functional analysis, hsa-miR-100, hsa-miR-193a-3p and hsa-miR-193a-3p mimics and inhibitors and non-targeting miRNA mimic and inhibitor controls from Thermo-Scientific (Supplementary Table 2) were transfected using Lipofectamine RNAiMAX (Invitrogen) according to the manufacturer's instructions. In brief, cells were plated in 6 cm diameter cell culture dishes to 60% confluence. For each dish, 20 pmol of mimics or inhibitors and 6 μl of transfection reagent were added into 1.5 ml of antibiotic-free opti-MEM medium (Invitrogen), separately, and then mixed together for forming the transfection complex. The transfection complex was added to cells and incubated for 24 h before replacing with complete medium. Overexpression or inhibition of miRNA after transfection was maintained for at least 10 days (assessed by real-time-PCR, not shown). Co-transfection of the mimic/inhibitor and plasmid DNA was conducted using Lipofectamine 2000 (Invitrogen). All transfections were performed according to the manufacturer's protocol.

### Pharmacological tests

Basal and transfected cell lines were plated into 96-well plates and treated, in triplicate, with different doses of rapamycin (Cell Signaling), from 0.1 nmol/liter to 500 nmol/liter; for 72 h. Cell proliferation was assessed by colorimetric Cell Proliferation Reagent WST-1 (Roche Applied Science), following the supplied protocol. The absorbance was determined using a microplate reader (iMARK microplate reader, Biorad) at a test wavelength of 450 nm. The variation in drugs sensitivity was analyzed calculating IC50 value. Triplicate wells for each condition were examined, and each experiment was repeated in triplicate.

### Cell proliferation and wound healing assay

H727 cells transfected with mimic or inhibitor miR-100 and with controls were plated into 96-well plates and after 0, 24, 48, 72, 96 h after transfection, 10 μl of WST-1 salt (Roche) were added to each well. Plates were incubated for 1 h before measuring the absorbance at 450nm in a microplate reader (Biorad). For wound healing assay transfected cells were plated into 6-well plates. After the cells reached sub-confluence, wounds were created using a 200-μl pipet tip. The cells were rinsed with medium to remove any free-floating cells. Cells were cultured at 37°C and wound healing was observed at different time points and photographed (magnification, ×100). Duplicate wells for each condition were examined, and each experiment was repeated in triplicate.

### Apoptosis assay

H727 cells with or without miR-100 over-expression (see Results section) were exposed to 1 μM and 10 μM rapamycin for 48 hours and then collected. Apoptosis was measured by staining the cells with Annexin V-FITC and PI using PE Annexin V apoptosis detection kit (BD pharmingen) and analyzed using using BD FACSCalibur flow cytometer.

### Western blot analysis

Whole protein extracts from normal and transfected cells were prepared in RIPA lysis buffer (Sigma) supplemented with 1% protease and 1% phosphatase inhibitor cocktail (Complete; Roche Diagnostic). For each experiment, protein concentration was determined using BCA protein assay kit (Pierce), and 50 μg of protein were resolved on a 8% SDS-PAGE gel and transferred to nitrocellulose membranes. Blots were blocked with 5% BSA in Tris-buffered saline-Tween 0.1% and incubated overnight at 4°C with mTOR (1:1000; clone L27D4, Cell Signalling), phospho-mTOR (1:1000; clone 59.S2448, S2448, Santa Cruz), phospho-p70S6K (1:1000; clone 1A5, T389, Cell Signalling), phospho-AKT (1:1000; polyclonal, S473, Santa Cruz), phospho-NDRG1 (1:1000; polyclonal, T346, Cell Signalling) and vinculin (1.1000; clone 7F9, Santa Cruz). Immuno-reactive proteins were visualized using horseradish peroxidase-conjugated anti-mouse antibody and enhanced chemi-luminescence substrate (Amersham) and images acquired with Chemi-doc (Biorad). The optical density of the appropriately sized bands was measured using the ImageJ free-software (http://rsbweb.nih.gov/ij/).

### Luciferase assay

Luciferase reporter construct was PCR-amplified from the wild-type 3ʹUTR of human mTOR mRNA containing the putative binding sites for miRNAs and inserted into the *XhoI* and *NotI* sites downstream of the stop codon of *Renilla* luciferase in psiCHECK 2 vector (Promega) resulting in the psiCHECK 2_3ʹmTOR plasmid. Plasmid sequence was verified by using Sanger method (data not shown). HEK-293T cells were grown in a 48-well plate were co-transfected with 12.5 nM of either NC or miRNAs duplex, 20 ng of psiCHECK-2 or psiCHECK-2_3ʹ-mTOR reporter plasmids. Luciferase assay was performed according to the manufacturer protocol (Promega). The psiCHECK-2 scramble-vector and siRNA anti-renilla were used as negative and positive control, respectively. Transfection was performed in duplicate and was repeated at least three times in independent experiments.

### Statistical analysis

Tissue and *in vitro* data were analyzed using appropriate statistical tests (Chi-square, Student's t, ANOVA and Spearman tests). Carcinoid patients only were used for survival analyses and follow up data were available in 69 cases. Kaplan–Meier survival times were calculated, and subgroups were compared by the log-rank test. Median miRNA expression levels were used as cut-offs. Multivariate Cox regression model was used to assess the association of different clinical, pathological and molecular variables with time to progression. Due to the low number of deaths overall survival analysis was not performed. IBM SPSS statistica software version 22 (IBM corporation, Armonk, USA) was used and *p* value of less than 0.05 was considered statistically significant.

## SUPPLEMENTARY MATERIALS FIGURES AND TABLES


