# Supplementary material for: High miR-100 expression is associated with aggressive features and modulates TORC1 complex activation in lung carcinoids
Source: Oncotarget. 2018 Jun 8;9(44):27535–46. doi: 10.18632/oncotarget.25541 (PMC6007959; doi:10.18632/oncotarget.25541)
Supplement: Supplementary file 1 [file oncotarget-09-27535-s001.pdf]

High miR-100 expression is associated with aggressive features and modulates TORC1 complex activation in lung carcinoids

SUPPLEMENTARY MATERIALS

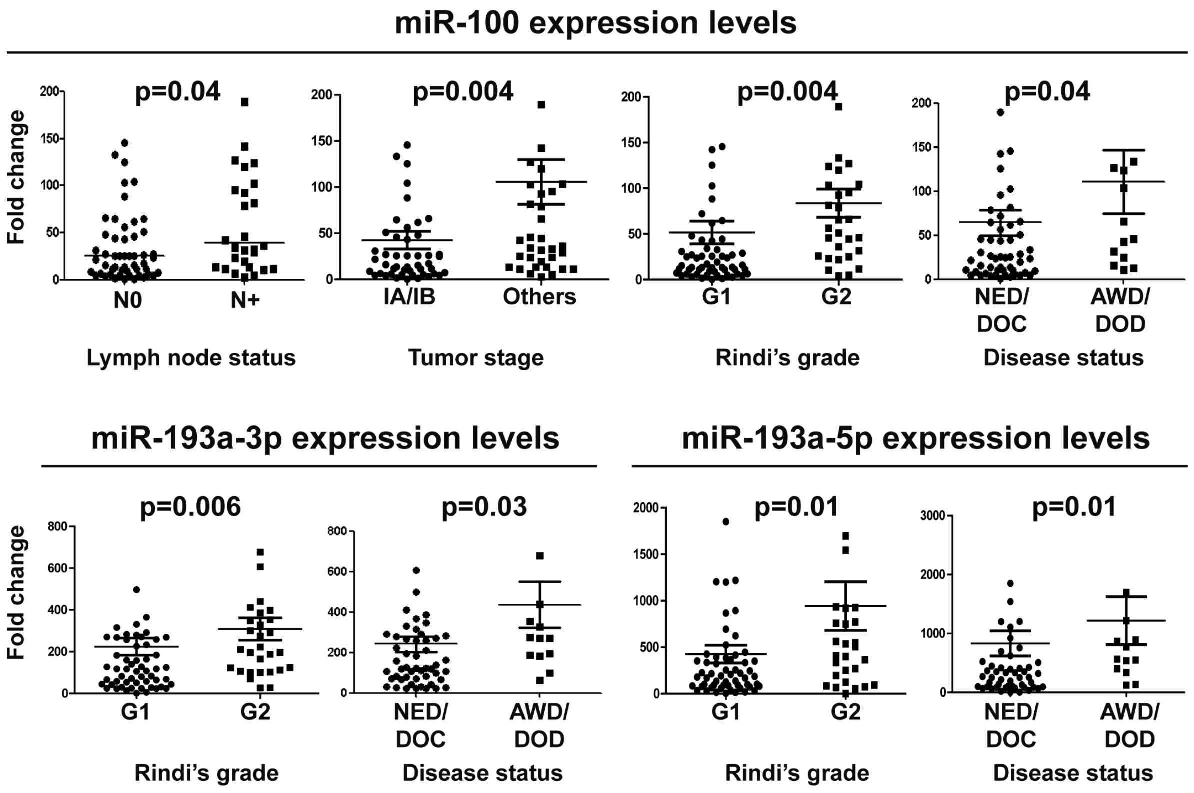

Supplementary Figure 1: Correlation of miR-100, miR-193a-3p and miR-193a-5p expression levels with clinical and pathological characteristics in lung carcinoids.

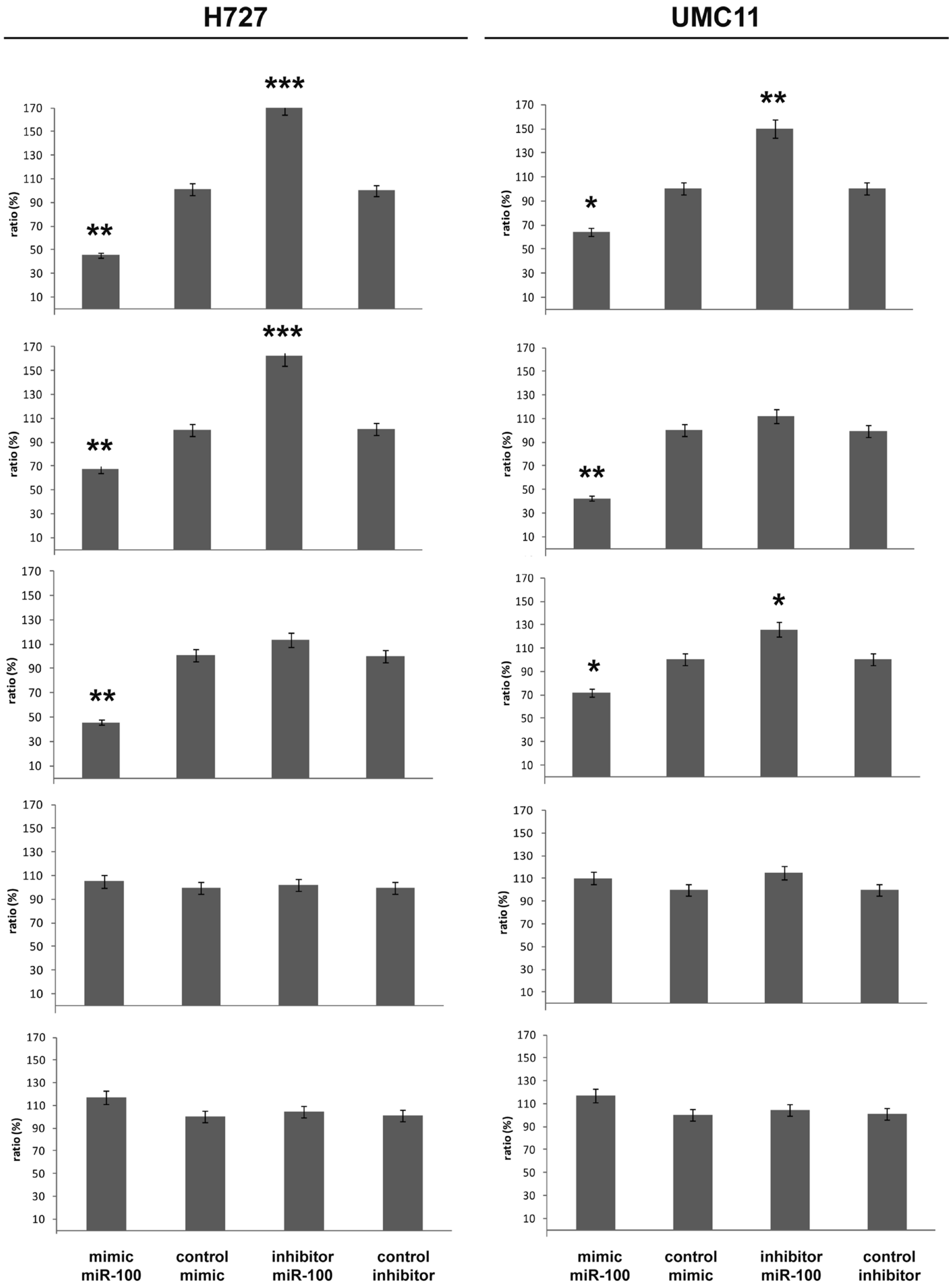

Supplementary Figure 2: Quantification of Western blot bands illustrated in Figure 2G and 2H, by means of ImageJ software Data was represented as mean ± SEM. $^{*}p < 0.05$, $^{**}p < 0.01$, $^{***}p < 0.001$.

Supplementary Table 1: Description of case series of 142 lung neuroendocrine neoplasms

	TC<br>#50	AC<br>#42	LCNEC<br>#29	SCC<br>#21
**Sex (M/F)**	21/29	23/19	26/3	16/5
**Age (mean)**	55	58	65	66
**Tumor stage pT1/pT2/pT3-4**	35/11/4	20/16/5	6/16/3	3/6/0
**Size (median)**	2.8	4.07	3.5	3.2
**Nodal status N0/N+**	35/15	22/20	18/11	18/3
**Proliferation index (mean)**	4.5	9.5	66.1	87.6

Supplementary Table 2: List of miRNA assays and genes used in real-time PCR and mimic/inhibitor miRNA used for cell transfection

miRNA/gene	***Assays code***
**hsa-miR99a-5p**	000435
**hsa-miR99b-5p**	000436
**hsa-miR100a-5p**	000437
**has-miR-155a-5p**	002623
**has-miR-193a-3p**	002250
**has-miR-193a-5p**	002281
**has-miR-199-3p**	002304
**RNU6B**	001093
**mTOR**	Hs-00234508_m1
**ACTB**	Hs-01060665_g1
Mimic/inhibitor	***Assays code***
**hsa-miR100a-5p mimic**	MC10188
**hsa-miR100a-5p inhibitor**	MH10188
**has-miR-193a-3p mimic**	MC11123
**has-miR-193a-3p inhibitor**	MH11123
**has-miR-193a-5p mimic**	MC11786
**has-miR-193a-5p inhibitor**	MH11786
**Mimic negative control**	4464058
**Inhibitor negative control**	4464076
